# Supplementary material for: GRT-R910: a self-amplifying mRNA SARS-CoV-2 vaccine boosts immunity for ≥6 months in previously-vaccinated older adults
Source: Nat Commun. 2023 Jun 6;14:3274. doi: 10.1038/s41467-023-39053-9 (PMC10242235; doi:10.1038/s41467-023-39053-9)
Supplement: Supplementary file 1 — Supplementary Information [file 41467_2023_39053_MOESM1_ESM.pdf]

Supplementary Figure S1: IgG and neutralizing antibody GRT-R910 samRNA dose comparison (10µg versus 30µg) and assay correlations

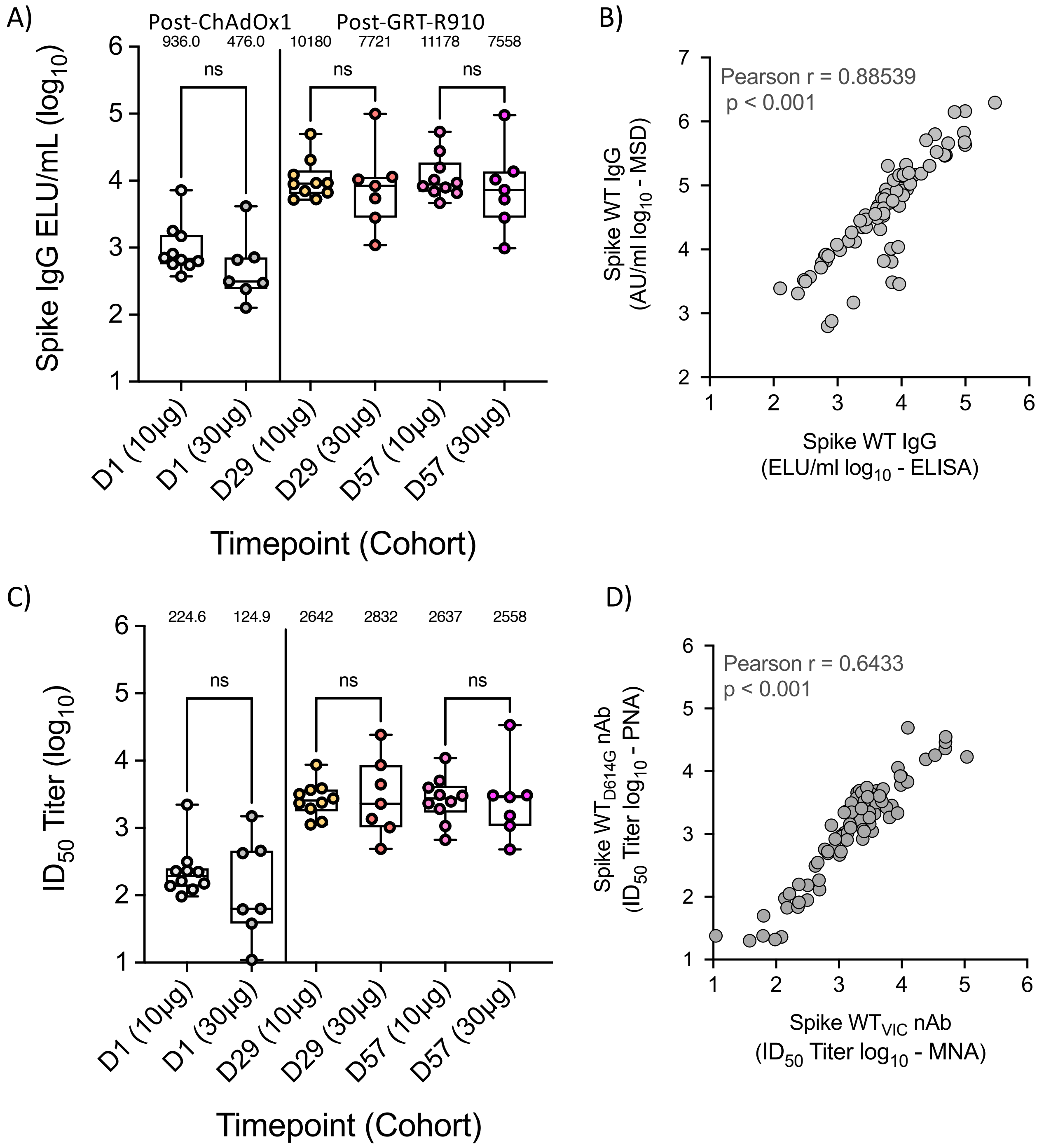

Supplementary Figure S1:

A) & B) Levels of Spike_{WT} IgG assessed by ELISA. Box and Whisker plots (min-max; median) show IgG levels in ELISA Units per ml (ELU/ml). Geometric mean (GeoMean) for each treatment day is indicated. A) Comparative IgG levels from participants in cohort 1 (n=10; 10µg GRT-R910) and cohort 2 (n=7, 30µg GRT-R910) at treatment days D1 (p=0.1398), D29 (p=0.6691), and D57 (p=0.5362). Statistical significance as analyzed by two-tailed Mann-Whitney test for each comparison is indicated (n.s.: not significant; p>0.05). B) Pearson correlation coefficient (r) of all Spike_{WT} IgG data points (n=87; p<0.001) shown in Figure 2 B) and C) assessed by ELISA (ELU/ml) and MSD in arbitrary units per ml. C) & D) Levels of Spike_{VIC} (Victoria/01/2020 strain; Spike_{VIC}) neutralizing antibody (nAb) titers assessed by microneutralization assay (MNA). Box and Whisker plots (min-max; median) show neutralization ID_{50} titers. Geometric mean (GeoMean) for each treatment day is indicated. C) Comparative nAb ID50 titers levels from participants in cohort 1 (n=10; 10µg GRT-R910) and cohort 2 (n=7, 30µg GRT-R910) at treatment days D1 (p=0.4747), D29 (p=0.8868), and D57 (p=0.7396). Statistical significance as analyzed by two-tailed Mann-Whitney test for each comparison is indicated (n.s.: not significant; p>0.05). D) Pearson correlation coefficient (r) of all Spike ID_{50} data points (n=78; p<0.001) shown in Figure 3 B) and C) assessed by MNA (Spike_{VIC}) and pseudovirus neutralization assay (PNA; Spike_{D614G}).

Supplementary Figure S2: Nucleocapsid IgG responses for study participants, healthy pre-pandemic, and convalescent donors

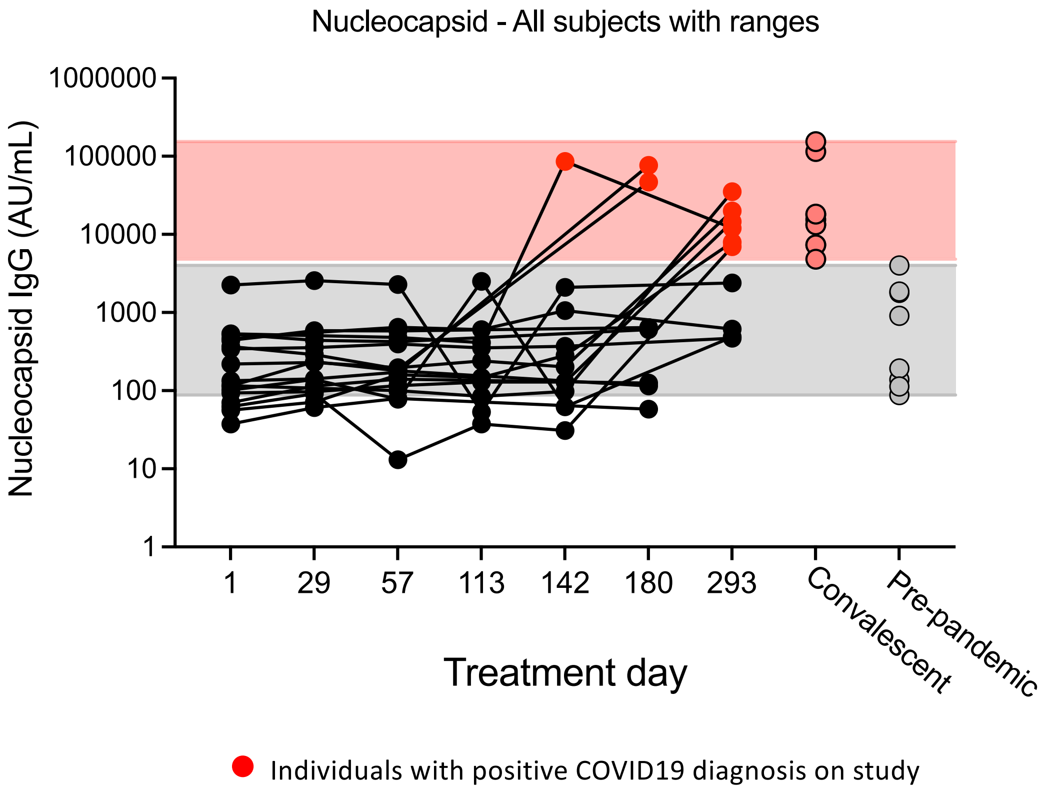

Supplementary Figure S2:

Levels of Nucleocapsid IgG assessed by MSD assay. Box and Whisker plots (min-max; median) show IgG levels in Arbitrary Units per ml (AU/ml). Longitudinal IgG levels from participants in cohorts 1 & 2 (n=17) from D1 through D293 (black circles). Sample-timepoints after SARS-CoV-2 diagnosis are indicated in red. Positive (convalescent) and negative (pre-pandemic) ranges are indicated in red and grey, respectively.

Supplementary Figure S3: Comparative Fold Change Analyses of IgG responses

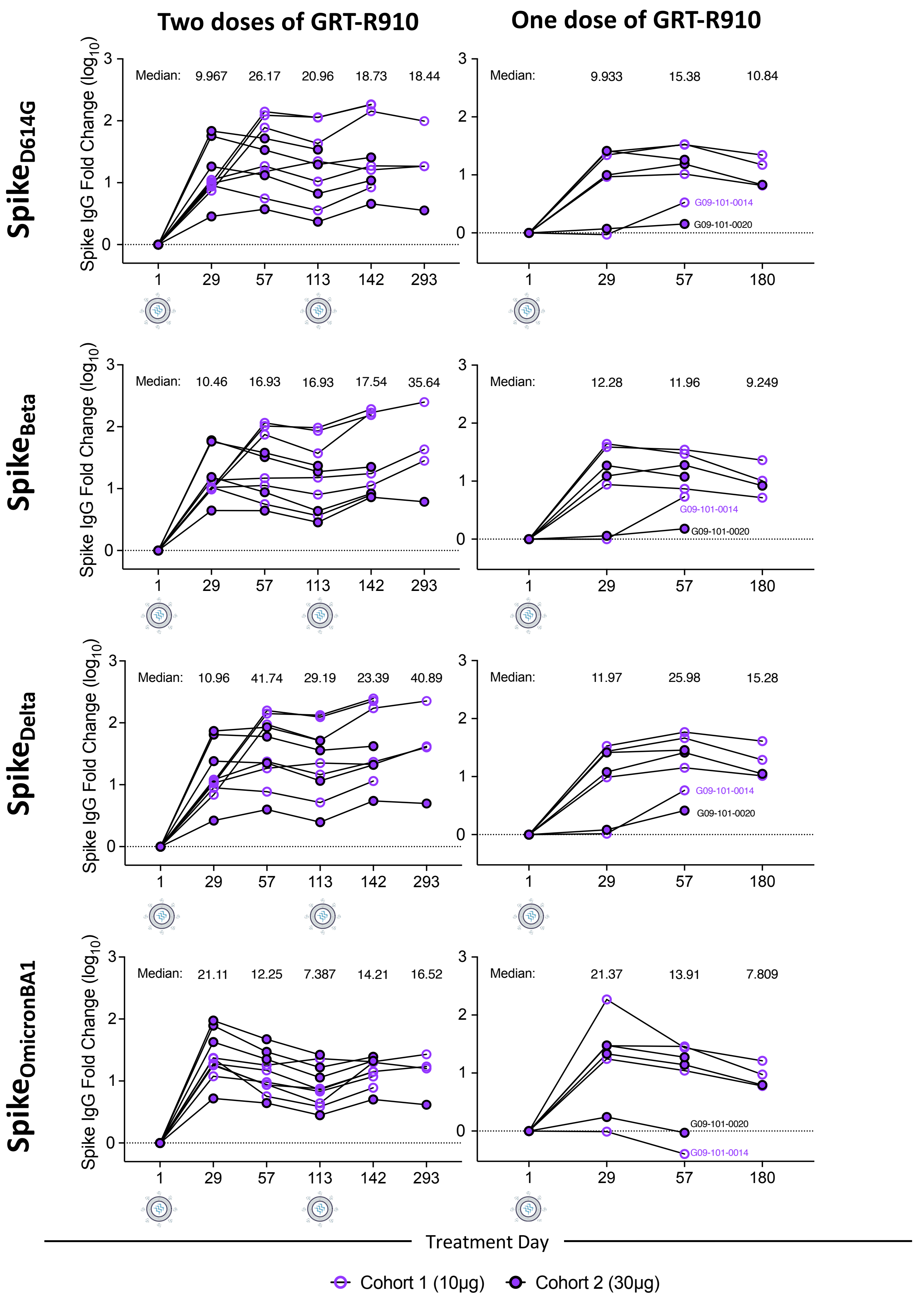

Supplementary Figure S3:

Levels of $Spike_{WT}$ and VOC $Spike_{Beta}$, $Spike_{Delta}$, and $Spike_{OmicronBA1}$ IgG assessed by MSD assay are shown as fold-change (log_{10}) over baseline (BL, 5-7 months post second AZD1222 vaccination) for each individual participant in cohort 1 (10µg dose, n=10; open circles) and cohort 2 (30µg dose, n=7, closed circles) who received two doses of GRT-R910 (n=10, left panels) or a single dose of GRT-R910 (n=7, right panels). Median fold-change levels over baseline for each timepoint are indicated. Corresponding summary statistics of the fold change over baseline are in Supplementary Table S2A.

Supplementary Figure S4: Comparative Fold Change Analyses of nAb responses

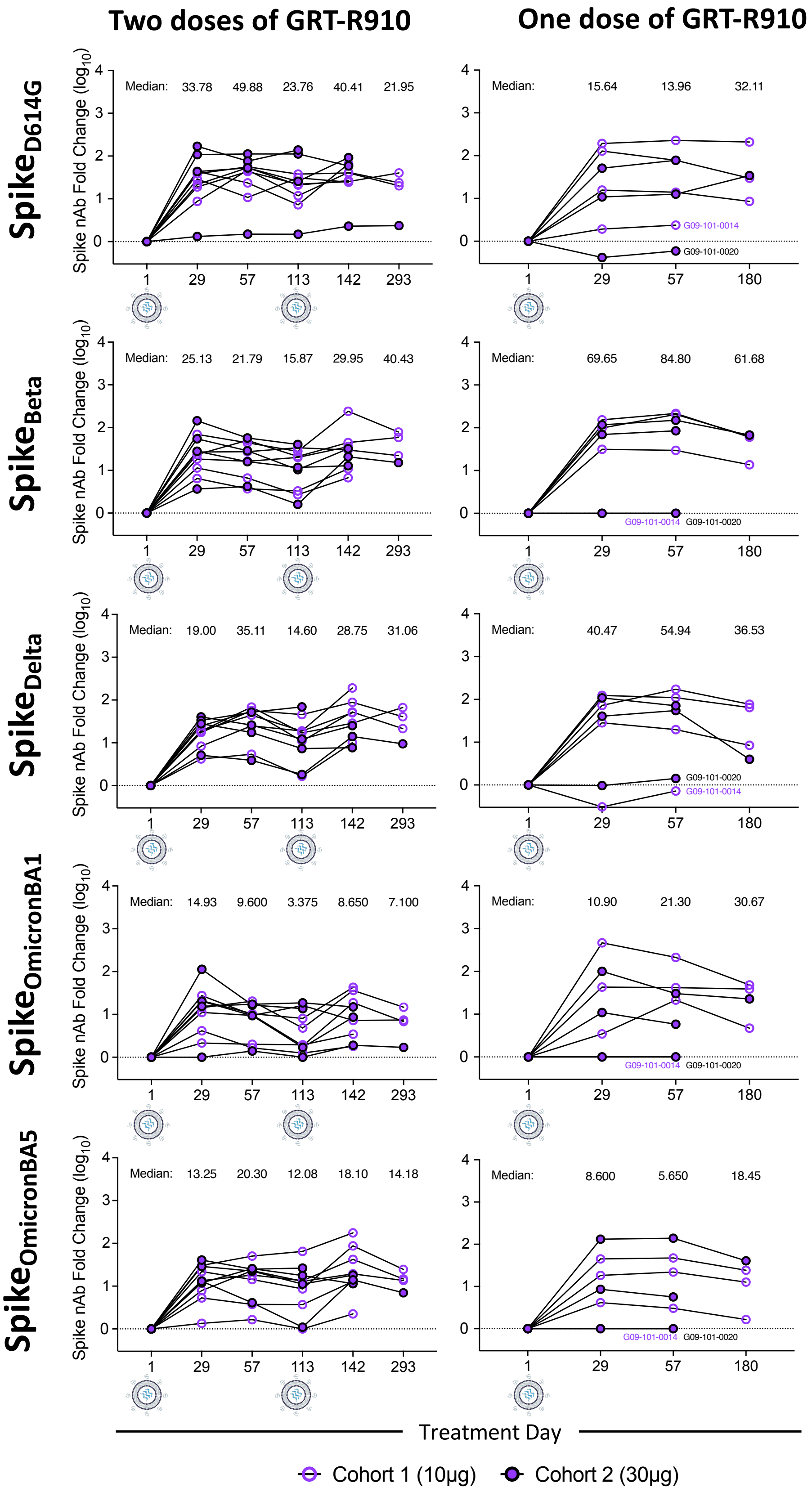

Supplementary Figure S4:

Levels of $Spike_{D614G}$, and VOC $Spike_{Beta}$, $Spike_{Delta}$, $Spike_{OmicronBA1}$, and $Spike_{OmicronBA5}$ neutralizing antibody (nAb) titers assessed by pseudovisus neutralization assay are shown as fold-change (log$_{10}$) over baseline (BL, 5-7 months post second AZD1222 vaccination) for each individual participant in cohort 1 (10µg dose, n=10; open circles) and cohort 2 (30µg dose, n=7, closed circles) who received two doses of GRT-R910 (n=10, left panels) or a single dose of GRT-R910 (n=7, right panels). Median fold-change levels over baseline for each timepoint are indicated. Corresponding summary statistics of the fold change over baseline are in Supplementary Table S2B.

Supplementary Figure S5: Spike-specific T cell responses are Th1-biased

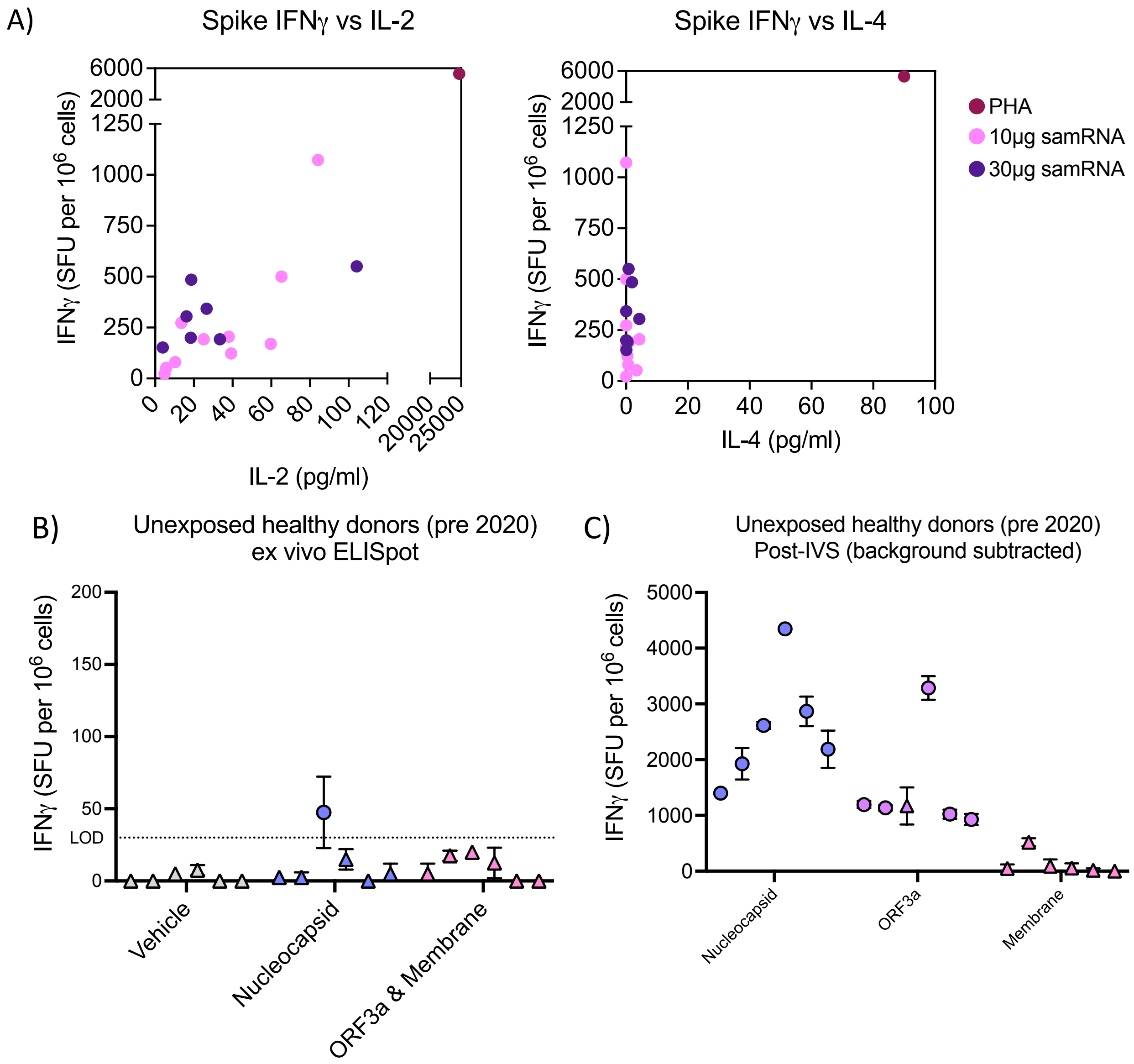

Supplementary Figure S5:

A) Th1 (IL-2) and Th2 (IL-4) cytokine levels in ELISpot supernatants assessed by MSD assay. X-Y plots showing corresponding ELISpot values (SFU/10^6 cells; Y-axis) and IL-2 (pg/ml; left graph) or IL-4 (pg/ml; right graph). Cohort 1 samples (n=10; pink), Cohort 2 (n=7; purple), and PHA control (burgundy) are shown. **B) & C)** T cell responses to TCE regions assessed by ELISpot in unexposed (pre-pandemic) donors. ELISpot data (mean +/- range for technical replicates) are shown in spot forming units per million PBMCs (SFU/10^6). Circles indicate positive (>LOD or >2xDMSO) responses, triangles indicate negative (<LOD or <2x DMSO) responses A) *Ex vivo* ELISpot (no antigen-specific expansion); B) Post-IVS ELISpot (post antigen-specific expansion).

Supplementary Figure S6: T cell responses to TCE epitopes in healthy pre-pandemic donors

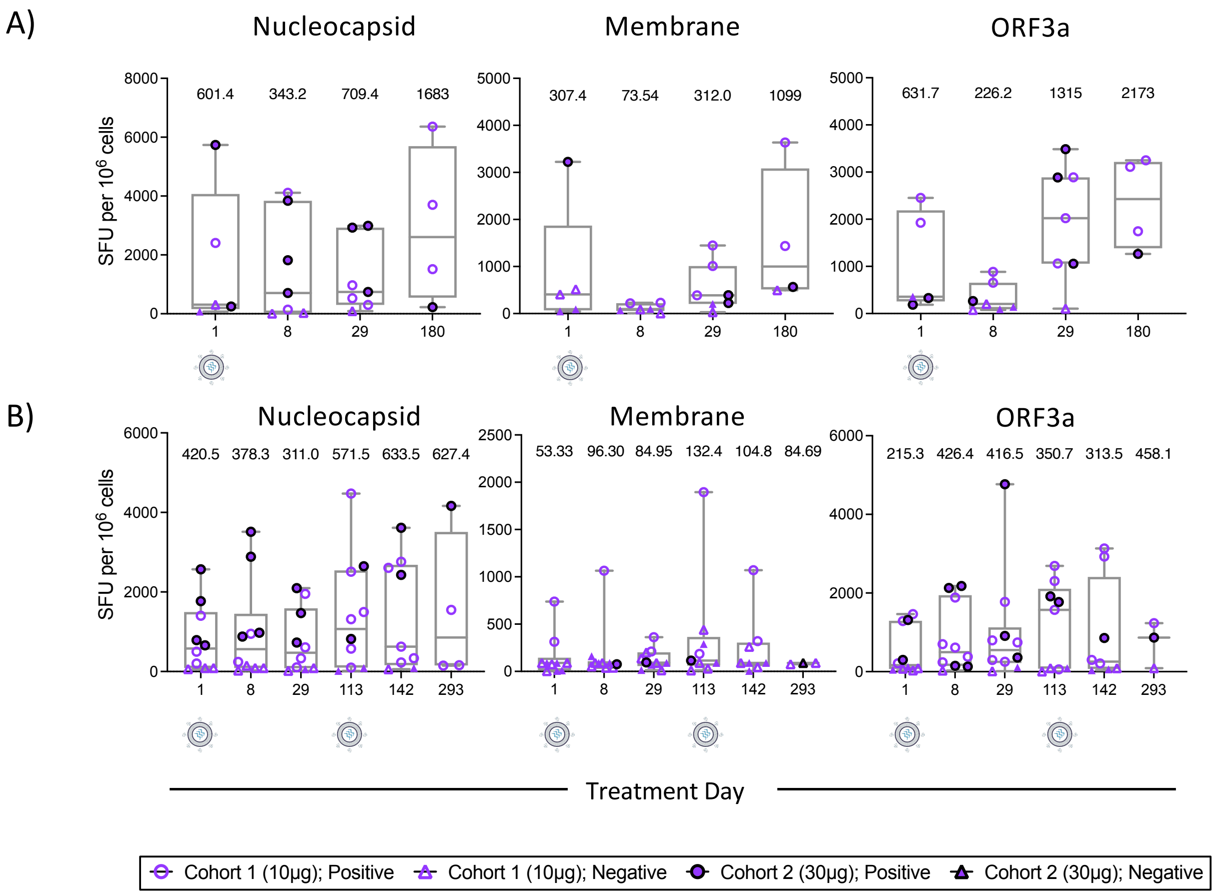

Supplementary Figure S6:

A) & B) T cell responses to overlapping peptide pools assessed by *ex vivo* IFNg ELISpot assay. Box and Whisker plots (min-max; median) show spot forming units per million PBMCs (SFU/10^6). GeoMean for each treatment day is indicated. Circles indicate positive (>LOD or >2xDMSO) responses, triangles indicate negative (<LOD or <2x DMSO) responses. Open and closed symbols indicate cohorts 1 and 2, respectively. A) T cell responses to TCE components are shown for participants in cohort 1 (n=4) and 2 (n=4) without optional second dose of GRT-R910 (total n=8) for treatment days 1 (n=5), D8 (n=7), D29 (n=7), and 180 (n=4). B) T cell responses to TCE components are shown for cohort 1 (n=6) and 2 (n=4) participants who received an optional second dose of GRT-R910 (total n=10) for treatment days D1 (n=10), D8 (n=10), D29 (n=10), D113 (n=10), D142 (n=9), and D293 (n=4).

Supplementary Figure S7: Intracellular Cytokine Staining (ICS) gating strategy

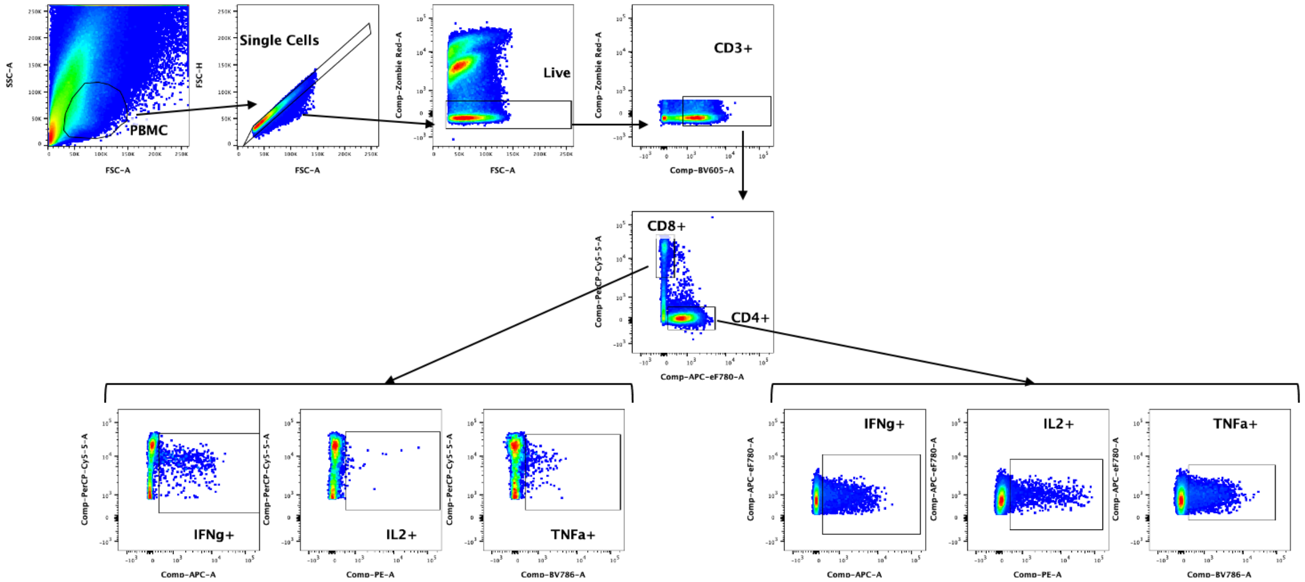

Supplementary Table and Dataset Legends:

Supplementary Table S1: Purchased serum samples
Details on serum samples purchases as assay controls (pre-pandemic and SARS-CoVo-2 convalescent) are shown.

Supplementary Dataset 1: IgG and nAb GeoMeans
Geometric mean values for IgG levels (ELU/ml or AU/ml for S_{WT}, W_{Beta}, S_{Delta}, $S_{OmicronBA1}$) and nAb titers (ID_{50} for for S_{WT}, W_{Beta}, S_{Delta}, $S_{OmicronBA1}$, $S_{OmicronBA5}$), are shown for subjects in cohorts 1 and 2 receiving 1 or two doses of GRT-R910 at various time points. Fold change values for D180/D29 and D293/D142 are shown.

Supplementary Dataset 2: Antibody Fold Change Statistics – IgG
Descriptive statistics of IgG fold change data are shown for subjects receiving 2 doses of GRT-R910 (Boosted individuals) and subjects receiving a single dose of GRT-R910 (Non-boosted individuals). Mean (+/- SD), Median, Q1, Q3, Min, Max are indicated for S_{WT}, W_{Beta}, S_{Delta}, and $S_{OmicronBA1}$ IgG levels (AU/ml).

Supplementary Dataset 3: Antibody Fold Change Statistics – nAb
Descriptive statistics of nAb fold change data are shown for subjects receiving 2 doses of GRT-R910 (Boosted individuals) and subjects receiving a single dose of GRT-R910 (Non-boosted individuals). Mean (+/- SD), Median, Q1, Q3, Min, Max are indicated for S_{WT}, W_{Beta}, S_{Delta}, $S_{OmicronBA1}$, and $S_{OmicronBA5}$ nAb titers (ID_{50}).

Supplementary Dataset 4: Peptides and pools
Peptides and pools utilized for T cell immune analyses are shown.

Supplementary Dataset 5: T cell SFU GeoMeans
Geometric mean values for ELISpot data (SFU/10^6 cells) for $Spike_{D614G}$ and TCE responses are shown for subjects in cohorts 1 and 2 receiving 1 or two doses of GRT-R910 at various time points. Fold change values for D180/D29 and D293/D142 are shown.

Supplementary Dataset 6: Pre-pandemic PBMC samples
Details on PBMC samples purchases or processed in-house before 2020 are shown for pre-pandemic donors.
