# Supplementary material for: GRT-R910: a self-amplifying mRNA SARS-CoV-2 vaccine boosts immunity for ≥6 months in previously-vaccinated older adults
Source: Nat Commun. 2023 Jun 6;14:3274. doi: 10.1038/s41467-023-39053-9 (PMC10242235; doi:10.1038/s41467-023-39053-9)
Supplement: Supplementary file 3 — Description to Additional Supplementary Information [file 41467_2023_39053_MOESM3_ESM.pdf]

Supplementary Table and Dataset Legends:

Supplementary Table S1: Purchased serum samples
Details on serum samples purchases as assay controls (pre-pandemic and SARS-CoVo-2 convalescent) are shown.

Supplementary Dataset 1: IgG and nAb GeoMeans
Geometric mean values for IgG levels (ELU/ml or AU/ml for S_{WT}, W_{Beta}, S_{Delta}, $S_{OmicronBA1}$) and nAb titers (ID_{50} for for S_{WT}, W_{Beta}, S_{Delta}, $S_{OmicronBA1}$, $S_{OmicronBA5}$), are shown for subjects in cohorts 1 and 2 receiving 1 or two doses of GRT-R910 at various time points. Fold change values for D180/D29 and D293/D142 are shown.

Supplementary Dataset 2: Antibody Fold Change Statistics – IgG
Descriptive statistics of IgG fold change data are shown for subjects receiving 2 doses of GRT-R910 (Boosted individuals) and subjects receiving a single dose of GRT-R910 (Non-boosted individuals). Mean (+/- SD), Median, Q1, Q3, Min, Max are indicated for S_{WT}, W_{Beta}, S_{Delta}, and $S_{OmicronBA1}$ IgG levels (AU/ml).

Supplementary Dataset 3: Antibody Fold Change Statistics – nAb
Descriptive statistics of nAb fold change data are shown for subjects receiving 2 doses of GRT-R910 (Boosted individuals) and subjects receiving a single dose of GRT-R910 (Non-boosted individuals). Mean (+/- SD), Median, Q1, Q3, Min, Max are indicated for S_{WT}, W_{Beta}, S_{Delta}, $S_{OmicronBA1}$, and $S_{OmicronBA5}$ nAb titers (ID_{50}).

Supplementary Dataset 4: Peptides and pools
Peptides and pools utilized for T cell immune analyses are shown.

Supplementary Dataset 5: T cell SFU GeoMeans
Geometric mean values for ELISpot data (SFU/10^6 cells) for $Spike_{D614G}$ and TCE responses are shown for subjects in cohorts 1 and 2 receiving 1 or two doses of GRT-R910 at various time points. Fold change values for D180/D29 and D293/D142 are shown.

Supplementary Dataset 6: Pre-pandemic PBMC samples
Details on PBMC samples purchases or processed in-house before 2020 are shown for pre-pandemic donors.
