# Supplementary material for: GRT-R910: a self-amplifying mRNA SARS-CoV-2 vaccine boosts immunity for ≥6 months in previously-vaccinated older adults
Source: Nat Commun. 2023 Jun 6;14:3274. doi: 10.1038/s41467-023-39053-9 (PMC10242235; doi:10.1038/s41467-023-39053-9)
Supplement: Supplementary file 11 — Reporting Summary [file 41467_2023_39053_MOESM11_ESM.pdf]

nature portfolio

Corresponding author(s): Karin Jooss

Last updated by author(s): May 16, 2023

Reporting Summary

Nature Portfolio wishes to improve the reproducibility of the work that we publish. This form provides structure for consistency and transparency in reporting. For further information on Nature Portfolio policies, see our Editorial Policies and the Editorial Policy Checklist.

Statistics

For all statistical analyses, confirm that the following items are present in the figure legend, table legend, main text, or Methods section.

n/a	Confirmed	
☐	☒	The exact sample size (*n*) for each experimental group/condition, given as a discrete number and unit of measurement
☐	☒	A statement on whether measurements were taken from distinct samples or whether the same sample was measured repeatedly
☐	☒	The statistical test(s) used AND whether they are one- or two-sided<br>*Only common tests should be described solely by name; describe more complex techniques in the Methods section.*
☒	☐	A description of all covariates tested
☒	☐	A description of any assumptions or corrections, such as tests of normality and adjustment for multiple comparisons
☐	☒	A full description of the statistical parameters including central tendency (e.g. means) or other basic estimates (e.g. regression coefficient) AND variation (e.g. standard deviation) or associated estimates of uncertainty (e.g. confidence intervals)
☐	☒	For null hypothesis testing, the test statistic (e.g. *F*, *t*, *r*) with confidence intervals, effect sizes, degrees of freedom and *P* value noted<br>*Give P values as exact values whenever suitable.*
☒	☐	For Bayesian analysis, information on the choice of priors and Markov chain Monte Carlo settings
☒	☐	For hierarchical and complex designs, identification of the appropriate level for tests and full reporting of outcomes
☒	☐	Estimates of effect sizes (e.g. Cohen's *d*, Pearson's *r*), indicating how they were calculated

Our web collection on statistics for biologists contains articles on many of the points above.

Software and code

Policy information about availability of computer code

Data collection	BD FACSDiva software version 9.2 (BD Biosciences)<br>AID ELISpot reader software version 7.0 (Autoimmun Diagnostika GmbH, Straßberg, Germany)<br>DISCOVERY WORKBENCH software version 4.0.13 (Meso Scale Discovery Inc., Rockville, MD, USA)<br>Methodical Mind software version 1.0 (Meso Scale Discovery)<br>SoftMax Pro software v6.5.1 (SpectraMax)
Data analysis	GraphPad Prism (GraphPad Software LLC, Version 9.0.1.)<br>FlowJo (BD, Version 10.6.1.)<br>SoftMax Pro software v6.5.1 (SpectraMax)<br>SAS version 9.4<br>R Studio version 1.3.1093

For manuscripts utilizing custom algorithms or software that are central to the research but not yet described in published literature, software must be made available to editors and reviewers. We strongly encourage code deposition in a community repository (e.g. GitHub). See the Nature Portfolio guidelines for submitting code & software for further information.

Data

Policy information about availability of data

All manuscripts must include a data availability statement. This statement should provide the following information, where applicable:

- Accession codes, unique identifiers, or web links for publicly available datasets
- A description of any restrictions on data availability
- For clinical datasets or third party data, please ensure that the statement adheres to our policy

Pseudonymized individual participant clinical data that underlie the results reported in this article are, to the extent permitted by applicable data protection laws, including the UK GDPR and the UK Data Protection Act, available for transfer. Interested investigators can obtain and certify the data transfer agreement (DTA) and submit requests to the principal investigator K.J. Principal investigator K.J. will reply to requests within 2 months. Investigators and institutions who consent to the terms of the DTA form, including, but not limited to, the use of these data for the purpose of a specific project and only for research purposes, and to protect the confidentiality of the data and limit the possibility of identification of participants in any way whatsoever for the duration of the agreement, will be granted access. Gritstone will then facilitate the transfer of the requested pseudonymized data. This mechanism is expected to be via a Gritstone Secure File Transfer Service, but Gritstone reserves the right to change the specific transfer method at any time, provided appropriate levels of access authorization and control can be maintained. Source data are, to the extent permitted by applicable data protection laws, including the UK GDPR and the UK Data Protection Act, provided with this paper.

Human research participants

Policy information about studies involving human research participants and Sex and Gender in Research.

Reporting on sex and gender	Information on participant sex and age are provided as part of subject demographics. The study design doesn't take into account sex/gender since it is not expected to see differences between the sex/gender with respect to the toxicity and tolerability in each small cohort of up to 10 subjects each. Informed consents used in this study does not allow us to share individual-level data, unless in the explicit case of an incident finding where sharing individual-level data favors subject's wellbeing.
Population characteristics	Eligible participants for cohorts 1 and 2 were ≥60 years of age of either sex who had received AZD1222 vaccine as part of a clinical trial or under the National Deployed Vaccine Programme in the UK. All participants were required to have completed their two-dose regimen of their primary vaccination series at least 2 months prior to study entry. Participants who had a history of prior confirmed SARS-CoV-2 infection or active infection were excluded from the study. The study design doesn't take into account sex/gender since it is not expected to see differences between the sex/gender with respect to the toxicity and tolerability in each small cohort of 10 subjects.
Recruitment	All 17 participants were enrolled in one site at Manchester University, UK. They were identified by the study team through a search on research registries where volunteers who are interested in taking part in research have self-registered and given their permission for their data to be shared with researchers, to be contacted about suitable studies. Such registries include information on those who received AZD1222 as a primary 2-dose series. Recruitment took place from consent-to-contact registries. The registries were established locally or nationally for COVID and COVID vaccinations, or more general registries where individuals have given consent to be contacted about such research. Once initial contact has been made researchers were responsible for seeking further consents as needed to cover participation in the trial itself. In addition, the site had several patients who had expressed interest previously onto their other COVID-19 vaccine trials but were not enrolled for various reasons (either the study closed to recruitment, or they did not meet the criteria for other studies, or they declined participation in such studies). All such potential participants have given consent to be contacted about future research opportunities. The use of such recruitment strategy is a usual approach for NIHR studies. On Sep/2021, Public Health recommendations started in the UK for booster doses which progressively impacted recruitment. Investigator expanded recruitment to other catchment areas through their local bulletin boards. As with any other non-randomized and non-controlled interventional trial, selection bias was not assessed and it's possible that only subjects who have a positive experience with AZD1222 vaccine and the participating in vaccines studies may have chosen to participate in GO-009.
Ethics oversight	The trial was conducted in accordance with the ethical principles derived from international guidelines, including the Declaration of Helsinki (7th revision, 2013), the international ethical guidelines of the Council for International Organizations of Medical Sciences, applicable Good Clinical Practice guidelines of the International Council for Harmonization, and all applicable laws and regulations. The trial protocol and all other relevant documentation were reviewed and approved by a local or central institutional review board or ethics committee for each site. All the participants provided written informed consent (with assistance from a legally authorized representative if required) before enrollment. All 3 sites on the study use the NSH Health Research Authority, London – West London & GTAC Research Ethics Committee. The sites are University Hospitals Birmingham NHS (Birmingham, United Kingdom), University Hospital of Leicester NHS Trust (Leicester, United Kingdom), Manchester University (Manchester, United Kingdom)

Note that full information on the approval of the study protocol must also be provided in the manuscript.

Field-specific reporting

Please select the one below that is the best fit for your research. If you are not sure, read the appropriate sections before making your selection.

☒ Life sciences ☐ Behavioural & social sciences ☐ Ecological, evolutionary & environmental sciences

Life sciences study design

All studies must disclose on these points even when the disclosure is negative.

Sample size

17 healthy participants 60 years or over who previously received a 2-vaccination series of AZD1222.
Sample size and power analysis for safety::
The goal of the safety evaluation for this study is to identify safety concerns associated with product administration. The ability of the study to detect SAEs can be expressed by the true event rate above which at least 1 SAE would likely be observed. For each group of 10 subjects, there is an 89% chance of observing at least 1 event if the true rate is 20% or higher. Binomial probabilities of observing at least 1 event among groups of size 10, 20, or 30 overall are presented in Table for a range of possible true adverse event rates. These calculations provide a more complete picture of the sensitivity of the study design to identify potential safety problems with the vaccine.

Table: Probability (%) of Detecting at Least 1 Adverse Event Under Different Incidence Rates

Incidence Rate (%)	Sample size = 10	Sample size = 20	Sample size = 30
0.01	0.10	0.20	0.30
0.1	1.00	1.98	2.96
1.0	9.56	18.21	26.03
2.0	18.29	33.24	45.45
5.0	40.13	64.15	78.54
10.0	65.13	87.84	95.76
20.0	89.26	98.85	99.88

Data exclusions

Subject time points after confirmed positive SARS-CoV-2 diagnosis while on study were excluded from immunological analyses, as inclusion of post-COVID samples would preclude any conclusions about the immunogenicity of the vaccine platform. This was not pre-specified in the protocol.

Replication

Technical replicates (duplicates or triplicates) were performed for immunological assessments. Antibody assays were performed at multiple different dilutions. IgG and nAb levels were assessed by 2 different assays in 2 separate laboratories, confirming successful replication of results.

Randomization

This was not a randomized trial, since it is an open label study of GRT-R910 without control arm. Eligible participants consist of individuals ≥60 years of age for Cohorts 1, 2, 3, and 4 and ≥18 to ≤59 years of age for Cohorts 5 and 6 who have received AstraZeneca's COVID-19 vaccine (Cohorts 1 and 2), an adenoviral COVID-19 vaccine (Cohorts 3 and 5), or an mRNA based COVID-19 vaccine (Cohorts 4 and 6) as part of a clinical trial or under the national deployed vaccine program.

Blinding

This was not a blinded trial. Blinding was not implemented since it is an open label study of GRT-R910 without control arm.

Reporting for specific materials, systems and methods

We require information from authors about some types of materials, experimental systems and methods used in many studies. Here, indicate whether each material, system or method listed is relevant to your study. If you are not sure if a list item applies to your research, read the appropriate section before selecting a response.

Materials & experimental systems

n/a	Involved in the study	
☐	☒	Antibodies
☐	☒	Eukaryotic cell lines
☒	☐	Palaeontology and archaeology
☒	☐	Animals and other organisms
☐	☒	Clinical data
☒	☐	Dual use research of concern

Methods

n/a	Involved in the study	
☒	☐	ChIP-seq
☐	☒	Flow cytometry
☒	☐	MRI-based neuroimaging

Antibodies

Antibodies used

Flow antibodies:

Zombie Red Fixable Viability Kit; Cat# 423110 Lot B308306
CD3+: Brilliant Violet 605 anti-human CD3; Clone: OKT3 BioLegend Cat# 317322 lot# B344790
CD4 Monoclonal Antibody (RPA-T4), APC-eFluor 780 Antibody; ebioscience Cat# 47-0049-42 lot: 2044762
PerCP/Cyanine5.5 anti-human CD8a; Clone: RPA-T8 BioLegend Cat# 301032 lot: B326771
Alexa Fluor 647 anti-human IFNg; Clone: 4S.B3 BioLegend Cat# 502516 lot# B328716

Brilliant Violet 785 anti-human TNFa; Clone: MAb11 BioLegend Cat# 502948 lot # B326860
PE anti-human IL-2 clone MQ1-17H12 BioLegend Cat# 500307 lot# B325787

ELISpot antibodies:
Capture Ab: Anti-human IFN-γ mAb (1-D1K), unconjugated; Mabtech Cat# 3420-3 Lot # 112.1
Detection Ab: Anti-human IFN-γ mAb (7-B6-1), biotin; Mabtech Cat# https://www.mabtech.com/products/3420-6 Lot# 55.3

Validation

Manufacturer validation statements:
Biolegend: All products sold by BioLegend Inc. comply with the requirements of ISO 13485:2016. This includes products labelled as Research Use Only (RUO) or GMP Research Use Only (GMP RUO), Analyte Specific Reagents (ASRs), and In Vitro Diagnostics (IVDs) including CE-Marked and registered products in selected countries.
Source: https://www.biolegend.com/en-us/quality/quality-assurance-certificates

eBioscience (now part of ThermoFisher Scientific): As a leading provider of high-quality, validated antibodies, our products are used by researchers around the world. Our customers purchase with confidence, knowing that we stand behind the quality of our antibodies with the Invitrogen™ antibody performance guarantee. If an Invitrogen antibody does not perform in your experiment as described on our website or data sheet, we will replace the product at no cost to you, or if you prefer, we will provide you with a credit for future purchase. Our performance guarantee is valid for products purchased directly from Thermo Fisher Scientific or any of our authorized distributors.
Source: https://www.thermofisher.com/us/en/home/life-science/antibodies/antibody-performance-guarantee.html

Mabtech:
https://www.mabtech.com/products/anti-human-ifn-g-mab-1-d1k-unconjugated-3420-3
https://www.mabtech.com/products/3420-6
https://www.mabtech.com/quality

Eukaryotic cell lines

Policy information about cell lines and Sex and Gender in Research

Cell line source(s)

HEK293T (ATCC; CRL-3216)
Vero-E6 (ATCC, CRL-1586)

Authentication

No authentication was performed

Mycoplasma contamination

Cells were confirmed negative for mycoplasma

Commonly misidentified lines
(See ICLAC register)

No commonly misidentified cell lines were used in this study

Clinical data

Policy information about clinical studies
All manuscripts should comply with the ICMJE guidelines for publication of clinical research and a completed CONSORT checklist must be included with all submissions.

Clinical trial registration

NCT05148962

Study protocol

Study protocol was provided as part of supplemental materials

Data collection

Primary endpoints were assessed as follows:
Occurrence of solicited local reactogenicity signs and symptoms for 7 days after GRT-R910 vaccination. Occurrence of solicited systemic reactogenicity signs and symptoms for 7 days after GRT-R910 vaccination. Occurrence of unsolicited AEs for 28 days after last study-administered GRT-R910 vaccination. Change from baseline for clinical safety laboratory parameters 7 days after last study-administered GRT-R910 vaccination. Occurrence of SAEs and adverse events of special interest (AESIs), including potentially immune-mediated medical conditions (PIMMCs), medically attended adverse events (MAAEs), and new onset chronic medical conditions (NOCMCs), throughout the entire study after GRTR910 vaccination.

Secondary outcomes were assessed as follows:
Response rate, and magnitude of SARSCoV-2-specific antibody binding and neutralization in serum samples. Response rate, magnitude, and breadth of SARS-CoV-2 specific T-cells as assessed by interferon (IFN)-gamma enzyme-linked immunospot (ELISpot) in PBMC samples.

Clinical data was collected in an electronic data capture system database based on case report forms. Solicited AEs and unsolicited TEAEs are summarized as counts and percentages, and any AEs after the administration of GRT-R910 vaccination were coded by Medical Dictionary for Regulatory Activities (MedDRA) version 25.0. Enrollment for cohorts 1 and 2 opened in September 2021 and was completed in December 2021. Immunogenicity data through 6 months following last dose of GRT-R910 were generated and analyzed by the study sponsor or VisMederi Srl. (Sienna, Italy) using participants' serum or peripheral blood mononuclear cells.

Outcomes	The primary and secondary endpoints for safety and immunogenicity were pre-defined per the protocol. The statistical analysis plan was finalized prior to primary analysis.

Flow Cytometry

Plots

Confirm that:

☒ The axis labels state the marker and fluorochrome used (e.g. CD4-FITC).

☒ The axis scales are clearly visible. Include numbers along axes only for bottom left plot of group (a 'group' is an analysis of identical markers).

☒ All plots are contour plots with outliers or pseudocolor plots.

☒ A numerical value for number of cells or percentage (with statistics) is provided.

Methodology

Sample preparation	Intracellular Cytokine Staining (ICS)<br>IVS-expanded PBMCs were stimulated with either minimal (8-11mer) or long overlapping (15mer) peptides, 20% (v/v) H20 and 80% (v/v) DMSO (vehicle control; VWR) or PMA/Ionomycin cell stimulation cocktail (positive control; Affymetrix, Santa Clara, CA, USA) in the presence of anti-Human CD28/CD49d antibody (BD Biosciences, San Jose, CA, USA), BD GolgiStop (BD Biosciences), and Brefeldin A (BioLegend) over a period of 18h. Following overnight incubation, cells were stained with live/dead Zombie-Red (BioLegend) and surface marker antibodies (CD8-PerCP-Cy5.5, CD3-BV605, from BioLegend; CD4-APC-eF780 from eBioscience) prior to fixation and permeabilization with FIX & PERM™ Cell Permeabilization Kit (ThermoFisher, Waltham, MA, USA). Following permeabilization, cells were stained for intracellular cytokines using anti-human IFNg-APC, TNFa-BV785, and IL-2-PE antibodies (BioLegend) prior to data acquisition on a BD LSRFortessaTM flow cytometer (BD Biosciences).
Instrument	BD LSRFortessaTM flow cytometer (BD Biosciences)
Software	BD FACSDiva software version 9.2<br>FlowJo software version 10.6.1 (FlowJo, LLC, Ashland, OR, USA)
Cell population abundance	Population frequencies (percent of parent) are indicated.
Gating strategy	Gating strategies for human samples (Supplementary Figure S5) are as follows: Lymphocytes (SSC-A vs FSC-A), single cells (FSC-H vs FSC-A), viable cells (FSC-A vs LD-ZombieRed), CD3+ cells (FSC-A vs CD3-BV605), CD4+ and CD8+ (CD4-APCeF780 vs CD8-PerCP-Cy5.5), CD8+ or CD4+ cytokine+ cells (CD8-PerCP-Cy5.5 vs IFNg-APC, TNFa-BV786, IL-2-PE; CD4-APCeF780 vs IFNg-APC, TNFa-BV786, IL-2-PE). Polyfunctionality analyses were performed using Boolean gating (FlowJo) and graphed using GraphPad Prism 9.0.1. Results are represented as % positive cell populations (frequency of parent). Data shown as background subtracted where indicated.

☒ Tick this box to confirm that a figure exemplifying the gating strategy is provided in the Supplementary Information.
